# Comparative Proteomic Analysis of Two Barley Cultivars (*Hordeum vulgare* L.) with Contrasting Grain Protein Content

**DOI:** 10.3389/fpls.2016.00542

**Published:** 2016-04-25

**Authors:** Baojian Guo, Haiye Luan, Shen Lin, Chao Lv, Xinzhong Zhang, Rugen Xu

**Affiliations:** ^1^Jiangsu Key Laboratory of Crop Genetics and Physiology/Co-Innovation Center for Modern Production Technology of Grain Crops, Key Laboratory of Plant Functional Genomics of the Ministry of Education, Barley Research Institution of Yangzhou University, Yangzhou UniversityYangzhou, China; ^2^JiangSu Coastal Area Institute of Agricultural SciencesYancheng, China

**Keywords:** barley, grain protein content, two-dimensional electrophoresis, differentially expressed protein, mass spectrometry

## Abstract

Grain protein contents (GPCs) of barley seeds are significantly different between feed and malting barley cultivars. However, there is still no insight into the proteomic analysis of seed proteins between feed and malting barley cultivars. Also, the genetic control of barley GPC is still unclear. GPCs were measured between mature grains of Yangsimai 3 and Naso Nijo. A proteome profiling of differentially expressed protein was established by using a combination of 2-DE and tandem mass spectrometry. In total, 502 reproducible protein spots in barley seed proteome were detected with a pH range of 4–7 and 6–11, among these 41 protein spots (8.17%) were detected differentially expressed between Yangsimai 3 and Naso Nijo. Thirty-four protein spots corresponding to 23 different proteins were identified, which were grouped into eight categories, including stress, protein degradation and post-translational modification, development, cell, signaling, glycolysis, starch metabolism, and other functions. Among the identified proteins, enolase (spot 274) and small subunit of ADP-glucose pyrophosphorylase (spot 271) are exclusively expressed in barley Yangsimai 3, which may be involved in regulating seed protein expression. In addition, malting quality is characterized by an accumulation of serpin protein, Alpha-amylase/trypsin inhibitor CMb and Alpha-amylase inhibitor BDAI-1. Most noticeably, globulin, an important storage protein in barley seed, undergoes post-translational processing in both cultivars, and also displays different expression patterns.

## Introduction

Barley is the fourth largest cereal crop in the world, which is not only widely used for food and feed, but also used for malting and brewing. The former requires grain protein content (GPC) to be as high as possible, whereas malting barley requires GPC to be at the proper level (See et al., 2002; Clancy et al., 2003; Cai et al., 2013). In Europe, the acceptable protein content range for malting barley was 9.5–11.5% (Pettersson and Eckersten, 2007). Higher protein content levels resulted in excess extract yield, which could produce a beer with hazy appearance; while lower protein levels decreased enzyme activity (Weston et al., 1993; Eagles et al., 1995). In addition, a positive correlation has been observed between GPC and diastatic power (Wang et al., 2003; Cai et al., 2013).

Barley GPC revealed significant differences in different varieties. Cai et al. (2013) analyzed the GPC of 59 cultivated and 99 Tibetan wild barley accessions in 2008 and 2009. The results showed that the GPC ranged from 8.02 to 13.50% with a mean of 10.56% in 2008 and ranged from 8.28 to 14.45% with a mean of 10.87% in 2009. Tibetan wild barley was found to have a higher GPC than cultivated barley. Comparative GPC analysis performed in 10 two-rowed spring malting barley cultivars showed that Klages and Logan showed the highest (14.57%) and lowest (12.56%) GPC, respectively, (Qi et al., 2005). Remarkably, variation in the proportions of the individual B, C, and D hordeins was observed in barley cultivars (Howard et al., 1996; Qi et al., 2005). All the above results indicated that genetic background had significant effects on GPC.

Recently, Two-dimensional electrophoresis combined with tandem mass spectrometry (MS) have resulted in new insights into the molecular basis of grain filling and seed maturation in plants (Finnie et al., 2002; Ge et al., 2012; Guillon et al., 2012; Dong et al., 2014; Jiang et al., 2014). For example, two-dimensional gel electrophoresis was used for a time-resolved study of the changes in proteins that occur during seed development in barley with approximately 1,000 low-salt extractable protein spots detected on the two dimensional gels. Among which 19 protein spots were identified by using matrix-assisted laser-desorption ionization time of flight MS or nano-electrospray tandem MS/MS. Proteins were accumulated throughout grain filling and maturation stages, which is reported to be functional characteristics of barley cultivars (Finnie et al., 2002). Glutelin is a predominant compound in rice seed. Two-dimensional gel electrophoresis (2-DE) analysis revealed remarkable differences in protein profiles of the wild rice species and the two cultivated rice materials. A total of 35 different pattern of expression protein spots were found for glutelin acidic subunits, glutelin precursors and glutelin basic subunits in wild rice species. Among those, 18 protein spots were specific and 17 major spots were elevated (Jiang et al., 2014). Chinese bread wheat cultivars Jimai 20 and Zhoumai 16 with different quality properties were investigated by 2-DE and MALDI-TOF/TOF-MS. A total of 117 different pattern of expression protein spots representing 82 unique proteins, which included isocitrate dehydrogenase, triticin precursor, low-molecular-weight glutenin subunit, and replication factor C-like protein. Remarkably, Class II chitinase and peroxidase 1 with isoforms in developing grains were shown to be phosphorylated by Pro-Q Diamond staining and phosphorylated protein site prediction (Guo et al., 2012). Therefore, proteome analysis is a tool that can be used both to visualize and compare complex mixtures of proteins.

In the present study, we reported a comparative proteomics analysis between Yangsimai 3 (feed barley cultivar) and Naso Nijo (malting barley cultivar) by 2-DE and tandem MS. The main objectives were: (1) to obtain comparative information on proteins expression profiling between feed barley and malting barley; and (2) to identify potential candidate proteins that influence GPC and barley grain quality.

## Materials and Methods

### Plant Material and Growth Conditions

Barley cultivars Yangsimai 3 and Naso Nijo were used in this study. Yangsimai 3 is a Chinese landrace of feed barley, two-rowed, with a high GPC. Naso Nijo is a Japanese two-rowed malting barley cultivar with a low GPC. Two barley cultivars were planted at the Yangzhou University Experimental Farm in the autumn of 2013. Twelve seeds of each cultivar were planted 10 cm apart with 20 cm between rows. The mature seeds were harvested from the middle region of the main spikelet, and then the seeds were frozen in liquid nitrogen and stored in -80°C for protein extraction. Three biological replicates were undertaken with 100 seeds per genotype being used for protein extraction and GPC measurement for each replication.

### Grain Protein Content Measurement

The mature grains were dried to a constant weight at 80°C, and ground in a Cyclotec 1093 sample mill (Hoganas City, Sweden) and sieved through a 0.5 mm screen. The total nitrogen content in grain was quantified according to the Kjeldahl method by using FOSS Kjeltec ^TM^ 2300 (Foss Analytical AB, Sweden; Kjeldahl, 1883). GPC was calculated by using the following formula: GPC = Nitrogen content × 5.83 × 100% (Sun et al., 2013). Statistical analysis of the differences in aerial part traits between cultivars was performed by using Student’s *t*-test.

### Protein Extraction

A 100 seeds of each genotype were pooled and milled to powder in liquid nitrogen. Approximately 0.1 g of flour was added into 1 mL of extraction buffer (2% SDS, 10% glycerol, 50 mM DTT, 40 mM Tris-HCl, pH 6.8) at 4°C (Hurkman and Tanaka, 2007). From this step onward, all manipulations were carried out at or below 4°C. The detailed method was as follows: the flour was extracted with stirring for 30 min and the insoluble material was removed by centrifugation at 20,000 *g* for 30 min (Fullerton City, CA, USA). The supernatant containing protein fractions were precipitated with four volumes of cold acetone containing 0.07% DTT. After 2 h incubation at -20°C, the extracts were centrifuged at 18,000 *g* for 30 min at 4°C and the supernatant was discarded. Protein pellets were resuspended with cold 80% acetone containing 0.07% DTT, incubated for 1 h at -20°C before centrifuging at 18 000 *g* for 15 min at 4°C (Kim et al., 2013). This step was repeated five times and the protein pellet was freeze-dried under vacuum. Protein pellets were solubilized and incubated in a protein buffer [7 M urea, 2 M thiourea, 2% CHAPS (powder to solution, w/v), 0.5% IPG buffer (v/v; pH 4-7 and 6-11; Fairfield City, OH, USA) and 36 mM DTT (5.6 mg/mL)] at room temperature for 1 h, vortexed every 10 min. The mixture was then centrifuged (20,000 *g*) for 15 min, and the supernatant was collected. Protein concentration was determined by Bradford assay (Bradford, 1976) with bovine serum albumin (BSA) used as a standard, and the *R*^2^ of standard curve was 0.9974 (**Supplementary Figure S1**).

### Two-dimensional Gel Electrophoresis and Image Analysis

Seed protein extract (200 μg) was loaded onto a GE Healthcare 18 cm IPG strip with a linear gradient of pH 4–7 and pH 6–11 during strip rehydration overnight. IEF was conducted using IPGPhorII (Fairfield City, OH, USA) at 20°C for a total of 65 kVh. Equilibration of the strips was performed immediately with 10 mL of two types of SDS equilibration buffer for 15 min each. Buffer 1 contained 1.5 M Tris-HCl (pH 8.8), 6 M urea, 30% glycerol, 2% SDS, and 1% DTT, and buffer 2 contained 1.5 M Tris-HCl (pH 8.8), 6 M urea, 30% glycerol, 2% SDS, and 2.5% iodoacetamide. The second dimension SDS-PAGE gels (12.5% linear gradient) were run on an Ettan DALTsix (Fairfield City, OH, USA), 0.5 h at 2.5 W per gel, then at 12 W per gel until the dye front reached the gel bottom. Upon electrophoresis, the protein spots were stained with silver nitrate according to the instructions of the PlusOne^TM^ Silver Staining Kit for proteins (Fairfield City, OH, USA), which offered improved compatibility with subsequent mass spectrometric analysis. Briefly, gels were fixed in 40% ethanol and 10% acetic acid for 30 min, and then sensitized with 30% ethanol, 0.2% sodium thiosulfate (w/v), and 6.8% sodium acetate (w/v) for 30 min. Then gels were rinsed with distilled water three times; 5 min duration each time; then incubated in silver nitrate (2.5 g/L) for 20 min. Incubated gels were rinsed with distilled water and developed in a sodium carbonate solution (25 g/L) with formaldehyde (37%, w/v) added (300 μL/L) before use. Development was stopped with 1.46% EDTA-Na_2_∙2H_2_O (w/v), and gels were stored in distilled water until they could be processed and reproducible spots were removed from them. Gel images were acquired using Labscan (Fairfield City, OH, USA). Image analysis was carried out with Imagemaster 2D Platinum Software Version 7.0 (Fairfield City, OH, USA). Three biological replicates of silver stained gels showed high reproducibility (>95%) by comparison using the Imagemaster 2D Platinum Software 7.0. Spot detection was performed automatically by the software used with the parameters smooth, minimum area, and saliency set to 2, 15, and 8, respectively, followed by manual spot editing, such as spot deletion, spot splitting, and merging. All the gels were matched to the reference gel in automated mode with Imagemaster 2D Platinum Software 7.0. The volume of each spot from three replicate gels was normalized and quantified against total spot volume in the Imagemaster 2D Platinum Software 7.0. Sequential k-nearest neighbor methods was used to impute missing values. Changes in the normalized spot volumes between experimental and control images were evaluated with a mixed linear mode. The spot number and normalized spot volume data were formatted in Excel. Student *t*-test analysis of protein expression was performed between Yangsimai 3 and Naso Nijo, and only those protein spots with the fold changes more than 1.5 and significant at *p* < 0.05 were considered as differentially expressed proteins.

When comparing the different pattern of expression protein spots between Yangsimai 3 and Naso Nijo, both quantitative and qualitative differences were observed. The quantitative differences can be grouped into two categories: up-regulated or down-regulated protein spot in Yangsimai 3 compared with Naso Nijo. The qualitative differences can be grouped into two categories: (i) specific expressed in Yangsimai 3 (SEY), expression in Yangsimai 3 cultivar, but not in Naso Nijo cultivar; (ii) specific expressed in Naso Nijo (SEN), expression in Naso Nijo cultivar, but not in Yangsimai 3 cultivar. Student’s *t*-test (*p* < 0.05) was used to calculate significant differences in relative abundances of protein spot features in the Yangsimai 3 compared with Naso Nijo. Spots with reproducible and significant variations, at least 1.5-fold up-regulated or down-regulated, were considered quantitative differentially expressed proteins.

### In-gel Digestion of Proteins

Protein spots were excised manually and transferred to 1.5 mL microcentrifuge tubes, and proteins with lower abundance were removed from all the replicate gels to pool and digest in a single tube. Protein spots were destained twice with 30 mM potassium ferricyanide and 100 mM sodium thiosulfate, and then rinsed with 25 mM ammonium bicarbonate in 50% acetonitrile. Protein spots were dehydrated with 100% acetonitrile, dried under vacuum, and 10 μL trypsin (10 ng/μL) was added, imbibed 40 min on ice. Then protein spots were covered by using 25 μL 25 mM ammonium bicarbonate and incubated for 16 h at 37°C. The peptides were eluted by using 30 μL 0.1% TFA, shaken for 10 min, the digestion solution was transferred to a new 1.5 mL tube, and then the protein spots were eluted by using 70% v/v acetonitrile and 0.1% v/v trifluoroacetic acid twice, the digestion solution was then transferred to a new 1.5 mL tube once more, incorporating digestion solution, freeze-dried for 2 h, condensing the volume to 10 μL and stored in -80°C.

### Identification of Proteins by Mass Spectrometry

The digestion solution was spotted on an MALDI target plate (1.0 μL) twice and recrystallized CHCA matrix dissolved in 0.1% TFA/70% ACN (0.5 μL). Mass Standards Kit for Calibration of SCIEX MALDI-TOF Instrument (Foster City, CA, USA) was used for Mass assignment. Each sample spot was desalted with 0.01% TFA, and completely dried. Protein identification was conducted using an SCIEX MALDI TOF-TOF^TM^ 5800 Analyzer equipped with a neodymium. For the MS mode, peptide mass maps were acquired in positive reflection mode, and the 800–4,000 m/z mass range was used with 4,000 laser shots per spectrum. A maximum of 20 precursors per spot with a minimum S/N ratio of 20 were selected for MS/MS analysis in 2 kV Positive modes. The contaminant m/z peaks originating from trypsin auto-digestion, or matrix were excluded from MS/MS analysis.

A MS/MS results were analyzed by using ProteinPilot software (Foster City, CA, USA), and the results were searched using MASCOT software^1^. Matches to protein sequences from the Viridiplantae taxon (Green plants) in NCBInr database (updated 6 June 2014, 17893860 sequences) were considered acceptable if: (1) A protein score was obtained from MASCOT, which rates scores as significant if they are above the 95% significance threshold (*p* < 0.05); (2) At least two different predicted peptide masses matched the observed masses for an identification to be considered valid; (3) The coverage of protein sequences by the matching peptides should be higher than 5%; (4) A parent ion mass tolerance of ±0.2 Da and an MS/MS tolerance of ±0.1 Da; (5) Acetylation of the N-terminus, cysteine as carboxylamidomethyl cysteine, pyroglu formation of N-terminal Gln and methionine in an oxidized form were set as possible modifications. To understand the function of the proteins, the identified proteins were classified by using the MapMan ontology defined by Thimm et al. (2004).

### Quantitative Real-Time PCR

Total RNA was isolated from the 10 days after flowering (DAF), 20 DAF, 30 DAF and mature seed using a TaKaRa MiniBEST Plant RNA Extration Kit (Tokyo City, Japan). cDNA was generated from the RNA with M-MLV reverse transcriptase (Tokyo City, Japan). Specific primers for quantitative real-time PCR (qRT-PCR) analysis were listed in Supplementary Table S1. Specificity of primers was checked by using NCBI database ^2^, and the PCR products were sequenced (Supplementary Table S1). Reaction was carried out in 20 μL reactions system containing 10 mM Tris-HCl (pH 8.5), 50 mM KCl, 2 mM MgCl_2_, 0.4 μL DMSO, 200 mM dNTPs, specific PCR primers 10 pmol/μL, Taq DNA polymerase 1 U, SYBR GREEN I fluorescence dye 0.5 μL. qRT-PCR was performed in clear tubes using an Applied Biosystems ViiA^TM^ 7 Real-Time PCR System (Carlsbad City, CA, USA) as follows: 94°C for 5 min, followed by 40 cycles at 94°C for 30 s, 58°C for 30 s, 72°C for 30 s, and a final extension of 72°C for 5 min. Actin was used as an internal control. All reactions were run in triplicate, Ct values were determined by the Applied Biosystems ViiA^TM^ 7 software with default settings. Differences between the Ct values of target gene and Actin were calculated as ΔCt = Ct *_target gene_* – Ct *_Actin_*, and the relative expression levels of target genes were determined as 2^-ΔCt^. For each sample, PCR was performed with three biological replicates. The average values of 2^-ΔCt^ were used to determine difference in gene expression.

## Results

### The Variation of Grain Protein Content between Barley Cultivar Yangsimai 3 and Naso Nijo

**FIGURE 1:**
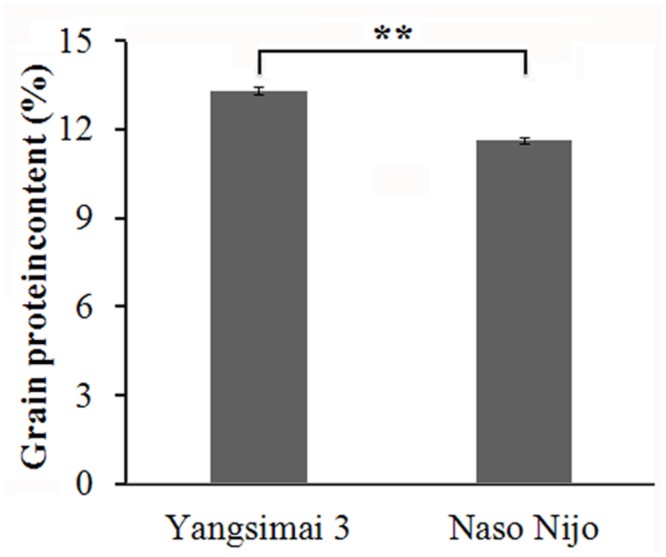
**Differences in grain protein content between two barley cultivars.** ^∗∗^*p* < 0.01.

The GPC of Yangsimai 3 and Naso Nijo are 13.3 and 11.6%, respectively (**Figure 1**), which indicates the GPC of Yangsimai 3 is 14.7% higher than Naso Nijo. Analysis shows the difference of GPC between the two cultivars is statistically significant (*p* < 0.01; **Figure 1**).

### Construction of a Differentially Expressed Protein Profiling of Grain Protein between Yangsimai 3 and Naso Nijo

**FIGURE 2:**
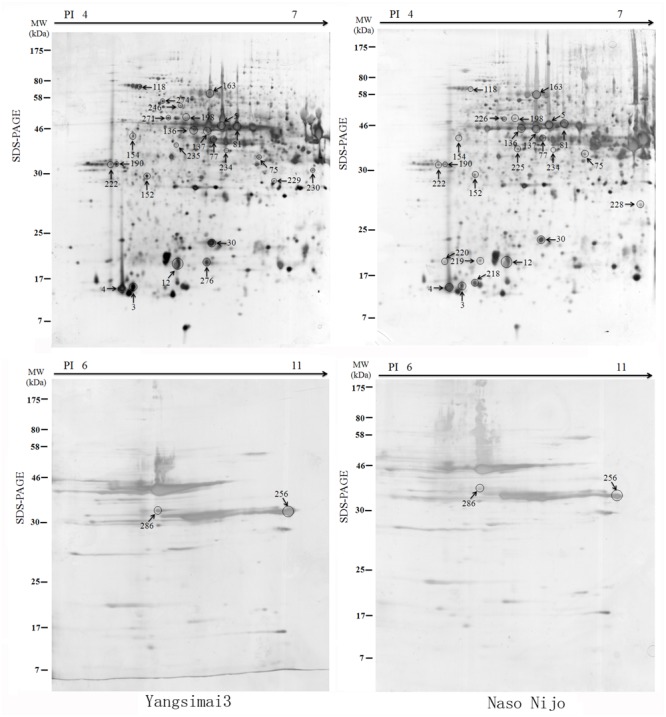
**The differentially expressed protein profiling between Yangsimai 3 and Naso Nijo**.

**FIGURE 3:**
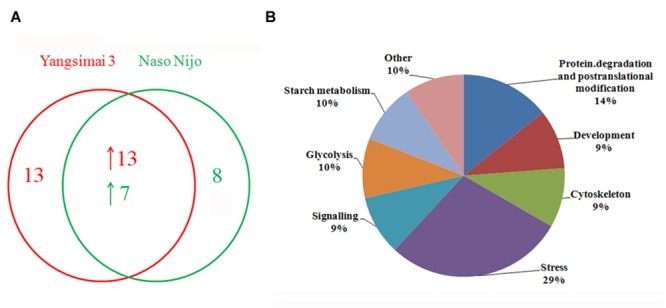
**The number of differentially expressed protein spots and protein function category. (A)** The number of differentially expressed protein spots; **(B)** Function category of differentially expressed proteins.

To construct a 2-DE map of barley grain proteins, grain proteins were separated by 2-DE with three biological replicates from Yangsimai 3 and Naso Nijo, respectively. At linear gradient of pH 4–7, 456, 464, and 448 protein spots were observed on 2-DE gels of Yangsimai 3, while 448, 460, and 452 protein spots were detected on 2-DE gels of Naso Nijo. At linear gradient of pH 6–11, 59, 55, and 57 protein spots were detected on 2-DE gels of Yangsimai 3, correspondingly, 58, 54, and 60 protein spots were detected on 2-DE gels of Naso Nijo (**Figure 2**; **Supplementary Figure S2**). Totally, 502 reproducibility protein spots were detected in both barley varieties, among which 41 (41/502, 8.17%) protein spots were found to be different pattern of expression between Yangsimai 3 and Naso Nijo by student’s *t*-test at *p* < 5%. When analyzing different pattern of expression between Yangsimai 3 and Naso Nijo, both quantitative and qualitative differences were observed. Student’s *t*-test was used to calculate significant differences in relative abundance of protein spots in the Yangsimai 3 compared with Naso Nijo. The qualitative differences can be grouped into two categories, which were SEY (specific expressed in Yangsimai 3; 13 entries) and SEN (specific expressed in Naso Nijo; eight entries). The quantitative differences can be grouped into up-regulated or down-regulated in Yangsimai 3, with 13 and 7 protein spots in each category, respectively (**Figure 3A**).

### Identification of Differentially Expressed Protein Spots

All differentially expressed protein spots between Yangsimai 3 and Naso Nijo were excised from representative 2-DE gels for identification, among which 34 protein spots were successfully identified by tandem MS, corresponding to 23 unique proteins (**Supplementary Figures S2** and **S3**). These identified protein spots can be further grouped into eight categories according to their biological functions, and the category with the most proteins identified was stress (6/23, 26.09%). The other categories referred to protein degradation and post-translational modification (three entries), development (two entries), cell (two entries), signaling (two entries), glycolysis (two entries), starch metabolism (two entries), and other function (four entries; **Figure 3B**).

**FIGURE 4:**
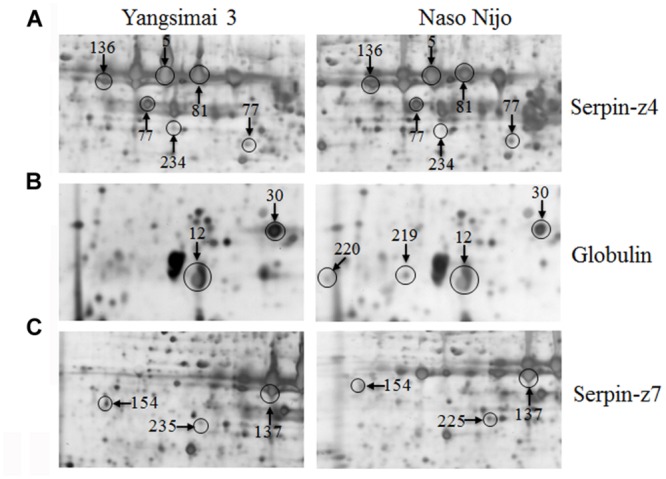
**Protein isoforms were observed on 2-DE gels.** Serpin-Z4 **(A)**, Globulin **(B)**, and Serpin-Z7 **(C)** isoforms which were observed on Yangsimai 3 and Naso Nijo 2-DE gels.

Further analysis revealed that these 34 identified protein spots derived from 23 different genes or gene families, among which 14 gel spots corresponding to 14 protein isoform identifications collapsed to three proteins (**Table 1**, Supplementary Table S2). For example, spot 12, 30, 219, and 220 identified as globulin (gi|167004); spot 137, 154, 225, and 235 identified as serpin-Z7 (gi|75282567); spot 5, 75, 77, 81, 136, and 234 identified as protein serpin-Z4 (gi|1310677). The isoforms matched to the same sequence, though they differed significantly with respect to their p*I*s and *Mr* (**Figure 4**). The number of isoforms for each protein ranged from 4 to 6. In the present study, four protein spots (spot 12, 30 219, and 220) were identified as globulin, which displayed an experimental Mr ranging from 20.380 to 22.147 kDa, less than the calculated Mr (637 aa, 72.551 kDa). MALDI-TOF MS/MS data which may be explained the Mr changes of the identified isoforms were summarized in Supplementary Table S2. All four protein spots were identified as C-terminal peptide sequences of globulin. In addition, some proteins have same protein name, but have different protein ID (Supplementary Table S2). For example, spot 190 (gi|22607) and 222 (gi|2492487) were identified as 14-3-3 protein, when aligned the two isoforms of 14-3-3 identified, it was shown that peptide 2 (LAEQAERYEEMVEFMEK) identified for gi|2492487 was common to both isoforms, whereas peptides 1 from each isoform (SAQDIALADLPTTHPIR and AAQEIALAELPPTHPIR) were unique and covered the same region of the proteins. Spot 198 (gi|326490934) and 274 (gi|326493636) were identified as enolase, both of them shared high homology (98.44%) and all of the peptides were common to both isoforms. Spot 9 (gi|326499406) and 118 (gi|326497219) were identified as heat shock cognate 70 kDa protein, both of them shared 88.28% homology between two isoforms, all of the identified peptides were common to both isoforms except peptide 2 (STAGDTHLGGEDFDNR) from gi|326497219 was unique. Spot 256 (gi|224386) and 286 (gi|255348352) were identified as B hordein protein (Supplementary Table S2), both of them shared 67.29% homology between two isoforms, peptide 1 (TLPMMCSVNVPLYR and TLPTMCSVNVPLYR) and 2 (VFLQQQCSPVPVPQR and VFLQQQCSPVAMSQR) were unique and cover the same region of the protein isoforms, other peptides were common to both isoforms. Spot 3 (gi|585290) and 218 (gi|123970) were identified as alpha-amylase inhibitor, both of them contain a conserved AAI domain, all of the identified peptide for each isoforms were unique for each isoforms.

**Table 1:** Identified differentially expressed proteins between Yangsimai3 and Naso Nijo in mature barley grains.

Spot no.	Protein name	Protein ID^a^	Experimental Mr(Da)/p*I*	Calculated Mr(Da)/p*I*	Species	Expression pattern^b^
**Cell**						
226	Actin	gi|326488133	42189/5.48	41849/5.23	*Hordeum vulgare*	SEN
228	Predicted protein	gi|297613620	27691/6.92	27303/4.93	*Oryza sativa*	SEN
**Development**						
229	Rab28	gi|326531218	31690/6.41	30439/5.23	*Hordeum vulgare*	SEY
12	Globulin	gi|167004	20412/5.45	72551/6.80	*Hordeum vulgare*	Up-regulated
30	Globulin	gi|167004	22147/5.72	72551/6.81	*Hordeum vulgare*	Up-regulated
219	Globulin	gi|167004	20386/5.23	72551/6.80	*Hordeum vulgare*	SEN
220	Globulin	gi|167004	20380/4.72	72551/6.80	*Hordeum vulgare*	SEN
**Glycolysis**						
198	Enolase	gi|326490934	42163/5.56	48601/5.39	*Hordeum vulgare*	Up-regulated
274	Enolase	gi|326493636	52435/5.31	48427/5.39	*Hordeum vulgare*	SEY
**Protein degradation and post-translational modification**						
5	Serpin-Z4	gi|1310677	40675/5.81	43307/5.61	*Hordeum vulgare*	Down-regulated
75	Serpin-Z4	gi|1310677	37625/6.24	43307/5.61	*Hordeum vulgare*	Down-regulated
77	Serpin-Z4	gi|1310677	38134/5.73	43307/5.61	*Hordeum vulgare*	Up-regulated
81	Serpin-Z4	gi|1310677	40580/6.01	43307/5.61	*Hordeum vulgare*	Up-regulated
136	Serpin-Z4	gi|1310677	40172/5.60	43307/5.61	*Hordeum vulgare*	Down-regulated
234	Serpin-Z4	gi|1310677	36214/5.92	43307/5.61	*Hordeum vulgare*	Up-regulated
137	Serpin-Z7	gi|75282567	40021/5.7	42851/5.45	*Hordeum vulgare*	Down-regulated
154	Serpin-Z7	gi|75282567	39041/4.92	42851/5.45	*Hordeum vulgare*	Up-regulated
225	Serpin-Z7	gi|75282567	36300/5.60	42851/5.45	*Hordeum vulgare*	SEN
235	Serpin-Z7	gi|75282567	37205/5.42	42851/5.46	*Hordeum vulgare*	SEY
230	Guanine nucleotide-binding protein subunit beta-like protein	gi|326491885	33456/6.90	36655/5.97	*Hordeum vulgare*	SEY
**Signaling**						
190	14-3-3 protein homolog	gi|22607	34126/4.77	29361/4.83	*Hordeum vulgare*	Up-regulated
222	14-3-3-like protein B	gi|2492487	34088/4.65	29787/4.67	*Hordeum vulgare*	Down-regulated
**Starch metabolism**						
163	Beta-amylase 1	gi|38349539	62321/5.70	57883/5.65	*Hordeum vulgare*	Down-regulated
271	Small subunit of ADP-glucose pyrophosphorylase	gi|27464770	42163/5.35	43861/4.91	*Hordeum vulgare*	SEY
**Stress**						
3	Alpha-amylase/trypsin inhibitor CMb	gi|585290	17054/4.91	17199/5.77	*Hordeum vulgare*	Up-regulated
4	CMd preprotein	gi|758343	16256/4.75	17894/5.24	*Hordeum vulgare*	Up-regulated
218	Alpha-amylase inhibitor BDAI-1	gi|123970	18374/5.20	17045/5.36	*Hordeum vulgare*	SEN
118	Heat shock cognate 70 kDa protein	gi|326497219	71675/5.13	72202/5.14	*Hordeum vulgare*	Up-regulated
9	Heat shock cognate 70 kDa protein	gi|326499406	70654/4.95	71629/5.09	*Hordeum vulgare*	Down-regulated
276	17 kDa class I small heat shock protein	gi|1536911	20653/5.68	16832/5.83	*Hordeum vulgare*	SEY
**Others**						
152	Lactoylglutathione lyase	gi|326493416	31.890/5.20	32811/5.34	*Triticum aestivum*	Up-regulated
246	ATP synthase beta subunit	gi|326492854	48524/5.51	59434/5.85	*Hordeum vulgare*	SEY
256	B hordein	gi|224386	31556/11.4	30850/8.26	*Hordeum vulgare*	Up-regulated
286	B hordein	gi|255348352	30782/7.55	30621/7.95	*Hordeum vulgare*	Up-regulated

*^a^Protein ID against the NCBI database. ^b^SEN, specific expressed in Naso Nijo; SEY, specific expressed in Yangsimai 3; Up-regulated: the abundance level of Yangsimai 3 is higher than Naso Nijo; Down-regulated: the abundance level of Yangsimai 3 is lower than Naso Nijo.*

### Expression Analysis of Three Differentially Expressed Proteins on RNA Level

**FIGURE 5:**
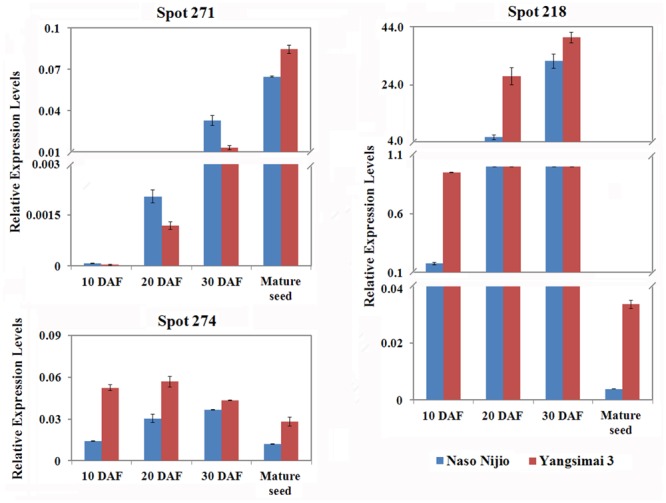
**Quantitative real-time PCR of mRNA expression patterns for selected protein spots in 10 DAF (days after flowering), 20 DAF, 30 DAF and mature seed**.

To investigate whether differentially expressed proteins between Yangsimai 3 and Naso Nijo were also observed on the RNA level, genes encoding three proteins (spot 274, 218, and 271) were selected for qRT-PCR analysis (**Figure 5**). As shown in **Figure 5**, the expression levels of gene encoding ADP-glucose pyrophosphorylase (spot 271) increased with maturity, enolase (spot 274) increased and then decreased in mature seed, Alpha-amylase inhibitor BDAI-1 (spot 218) increased from 10 to 30 DAF but then dramatically decreased in mature seed. Comparison the expression pattern of three genes encoding differentially expression protein between protein level and RNA level in mature seed, genes encoding enolase, and small subunit of ADP-glucose pyrophosphorylase displayed higher expressed in Yangsimai3 than Naso Nijo on the RNA level, but specific expression in Yangsimai 3 in protein level (**Figures 2** and **5**). Remarkably, gene encoding Alpha-amylase inhibitor BDAI-1 showed the opposite expression patterns on the protein level compared with RNA level (**Figures 2** and **5**).

### Integration with Genomic Information

To validate the potential implications of these functional annotations, the present results and those of a previous genetic analysis were integrated for GPC (Cai et al., 2013). Two proteins, including enolase and small subunit of ADP-glucose pyrophosphorylase (gi|326493636 and gi|27464770) were identified in the present study, both of which were mapped to on chromosomes 5^3^ and positioned 44.17 and 47.01 cM in barley, respectively. Remarkably, both proteins were closed to a QTL of controling barley GPC whose linkage marker was bPb-3412 at 45.6 cM on Chr. 5 (Cai et al., 2013).

## Discussion

Barley cultivars used for malt should have a GPC not exceeding 11.5% (Pettersson and Eckersten, 2007). Actually, GPC is greatly affected by the growing environment. For instance, stresses caused by levels of nitrogen, drought, and/or heat may increase the GPC in barley (Savin and Nicolas, 1996; Qi et al., 2006). Therefore, it is important to breed cultivars with lower and less environmentally influenced GPC (Bertholdsson, 1999). In the current study, Yangsimai 3 was used as feed barley with GPC of 13.3%, which displayed significant difference in GPC compared to Naso Nijo which was used as malting barley with GPC of 11.6%, according to the data from 2013. Thus, these two cultivars provide good studying material to perform comparative proteome analysis and discover potential protein candidates involved in stable GPC expression and regulation.

Grain protein content is also influenced by genetic factors in barley. Genome-wide association study (GWAS) was performed by using 59 cultivated and 99 Tibetan wild barley genotypes for identifing molecular markers associated with GPC. Results showed a total of 10 DArT markers (*p* < 0.01) were associated with GPC in barley (Cai et al., 2013). Further analysis indicated that *HvNAM* genes could play a role in controlling barley GPC (Cai et al., 2013). Variation in protein expression profiles of barley cultivars reflected genetic variations, which was illustrated by the identification of different alleles of β-amylase in two protein spots (Finnie et al., 2002). For example, Barke and Morex are two malting cultivars different in seed maturation days. Comparative proteomics analysis revealed that differentially expressed proteins reflected the faster maturation of Morex seeds (Finnie et al., 2006). In the current study, a differentially expressed proteins profiling of barley mature seed was constructed. Among the 502 protein spots that were reproducibly detected, a total of 34 differentially expressed protein spots were identified by tandem MS, which corresponded to 23 different proteins.

ADP-glucose pyrophosphorylase (AGPase) catalyzes the conversion of Glc-1-P and ATP to PPi and ADP-glucose, and is a key regulatory enzyme of starch biosynthesis (Preiss et al., 1991; Tetlow et al., 2004; Geigenberger, 2011). In barley, AGPase gene generates two transcripts, one of which encodes the cytosolic small subunits of ADP-glucose pyrophosphorylase in the endosperm and another encodes the plastidial SSU in leaves (Beckles et al., 2001). The *Risø16* mutant of barley lacks cytosolic AGPase activity in the endosperm, which leads to decreased grain weight. Also, *Risø16* mutant contained 90% total N and proteins of wild type at the transcriptional level, down-regulated enolase and beta-amylase 1 (Faix et al., 2012). In the present study, protein spot 274 and 271 were identified as enolase (gi|326493636) and small subunit of ADP-glucose pyrophosphorylase (gi|27464770), respectively, both of which were higher expressed in Yangsimai 3 than in Naso Nijo on the protein and RNA levels. Genomic information verified that the two proteins are close to a QTL for GPC (bPb-3412) on Chr. 5 (Cai et al., 2013). Integrating the above genomic results, the comparative expressions from our current study indicated that the specific expression of enolase (spot 274) and small subunit of ADP-glucose pyrophosphorylase (spot 271) in barley Yangsimai 3 could contribute to the GPC in barley seed.

Prolamins is the major storage proteins in barley seed protein, which is specifically synthesized in the starchy endosperm and divided into B, C, D and γ hordeins according to their mobility in SDS-electrophoretic gels (Kreis and Shewry, 1992; Piston et al., 2005). Among the four types of hordeins, B fraction accounts for 70–80% of the total prolamins content in most barley cultivars. Previous studies reported that B hordein was significantly correlated with GPC, B hordein content increased as the sowing date was postponed and was significantly affected by nitrogen levels (Qi et al., 2005, 2006). In the present study, two protein spots (spot 256 and 286) were identified as B hordein, and also displayed up-regulated in Yangsimai 3, which would contribute to the difference of GPC in mature barley seeds.

Serpins are the most abundant proteins in beer 2DE gels, and characterized by their function in malting barley (Jin et al., 2013). It was widely believed that serpins are beer foam-positive proteins and improve malt filterability (Maceda et al., 1991; Jin et al., 2013). In barley, serpin was approximately 43-kDa polypeptide, which irreversibly inhibits the endogenous and exogenous target proteinases (Roberts and Hejgaard, 2008). Comparative proteomics based on fluorescent difference gel electrophoresis (DIGE) was employed to quantitatively analyze proteins in cultivars Dan’er and Metcalfe in China, and the results showed that serpin Z4 and Z7 were the most remarkable differentially expressed proteins, which played an important role in malt filterability (Jin et al., 2013). Interestingly, mutiple protein isoforms of serpin Z4 and Z7 were observed in barley seed proteome (Finnie et al., 2002; Jin et al., 2013). In the present study, 10 protein spots were identified as serpin (serpin Z4 and Z7) and displayed multiple expression patterns (**Table 1**; Supplementary Table S2). In addition, globulin, which undergoes post-translational processing, is an important seed storage protein in cereal crops and functions in glycosylation and partial endoproteolytic cleavage (Heck et al., 1993; Herman and Larkins, 1999). In wheat, globulin-3 was cleaved post-translationally in embryos. Five major polypeptide spots of globulin-3 were identified by MS and N-terminal sequencing, and each spot displayed different Mr and pI, these post-translational processing events that lead to the maturation of the globulin family of proteins observed seed protein fraction that could be associated with type 1 diabetes or celiac disease following endoproteolytic processing (Koziol et al., 2012). In barley, globulin contains 637 amino acids with one signal peptide detected by the SignalP v4.0 program, the peptide starts at position 1 and ends at position 27. Four protein spots (spot 12, 30, 219, and 220) were identified as barely globulin in the current study, the MALDI-TOF MS/MS result revealed that the four protein spots only contains several fragments of the globulin, it is suggesting that globulin undergo cleavage of signal peptide from precursor, or non-specific degradation pathway.

The proteinaceous barley alpha-amylase/subtilisin inhibitor (BASI) is synthesized during grain filling and is an abundant protein of the endosperm and the aleurone layers of the mature seed (Mundy et al., 1983; Lecommandeur et al., 1987; Leah and Mundy, 1989). Recently, alpha-amylase inhibitor (BDAI-1) and CMb component of tetrameric alpha amylase inhibitor (CMb) were identified by using MS analysis. All of them were differentially expressed among four haze samples; further analysis indicated that BDAI-1, CMb were not predominant haze-active proteins, but growth factors of beer colloidal haze (Iimure et al., 2009). Two alpha-amylase inhibitors were identified in the current study, which were Alpha-amylase inhibitor BDAI-1 (spot 218) and CMb (spot 3) displayed specific expressed and down-regulated pattern in Naso Nijo, respectively. Dramatically, Alpha-amylase inhibitor BDAI-1 displayed higher expression in Yangsimai 3 than in Naso Nijo on the RNA level. These data suggested that different members in the protease inhibitor family may have different functions in barley cultivars, and also the mRNA of BDAI-1 maybe undergo post-transcriptional processing in the seed development. However, detailed studies on alpha-amylase inhibitor may facilitate a better understanding of the mechanisms involved in malting barley.

## Conclusion

In the present study, the GPC was significant difference in Yangsimai 3 and Naso Nijo. A differentially expressed profiling of grain proteins was established by using a combination of 2-DE and tandem MS. In total, 41 protein spots were detected differentially expressed between Yangsimai 3 and Naso Nijo, among which 34 protein spots corresponding to 23 different proteins were identified. In particular, differentially expressed proteins from the seed were mainly related to stress, protein degradation and post-translational modification, development, cell, signaling, glycolysis and starch metabolism. Seeds proteome explicitly displays that differentially expressed proteins are involved in GPC and malting quality. However, a malting barley cultivar is quite different from a feed barley cultivar and there is an interest to develop a greater knowledge of the determinants of malting and feed quality at the protein level, in order to improve the evaluation of new barley varieties. The quality of the barley is determined in part by the proteins content produced during grain filling, meanwhile the accumulation of storage proteins plays an important role in seed development by regulating the appearance of proteins in different development stages. Therefore, an understanding of the mechanism of how storage proteins accumulation initiates during seed development is required to be further investigated.

## Author Contributions

BG and HL carried out the seed proteins preparation, 2-DE analysis, mass spectrometry analysis and interpretation of the 2-DE data, and wrote portions of the manuscript. SL, CL, and XZ carried out image analysis and 2-DE data. BG and RX conceived of the project and wrote a draft of the manuscript and provided conceptual framework. All authors read and approved of the final manuscript.

## Conflict of Interest Statement

The authors declare that the research was conducted in the absence of any commercial or financial relationships that could be construed as a potential conflict of interest.

## Footnotes

1 http://www.matrixscience.com/

2 http://www.ncbi.nlm.nih.gov/tools/primer-blast/index.cgi?LINK_LOC=BlastHome

3 http://webblast.ipk-gatersleben.de/barley/

## Supplementary Material

FIGURE S1 **The standard curve for protein concentration determination**.

FIGURE S2 **All the replicates 2-DE gels in the present study**.

FIGURE S3 **Expression patterns for differentially expressed protein spots.** ^∗^*p* < 0.05, ^∗∗^*p* < 0.01.
