# Supplementary figures and images for: Comparative Proteomic Analysis of Two Barley Cultivars (Hordeum vulgare L.) with Contrasting Grain Protein Content
Source: Front Plant Sci. 2016 Apr 25;7:542. doi: 10.3389/fpls.2016.00542 (PMC4843811; doi:10.3389/fpls.2016.00542)

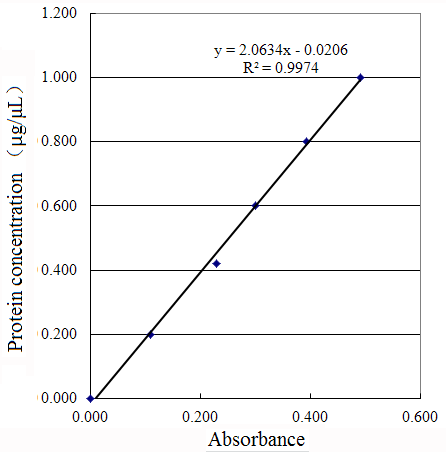

Supplement: FIGURE S1 — The standard curve for protein concentration determination. [file Image_1.TIF]

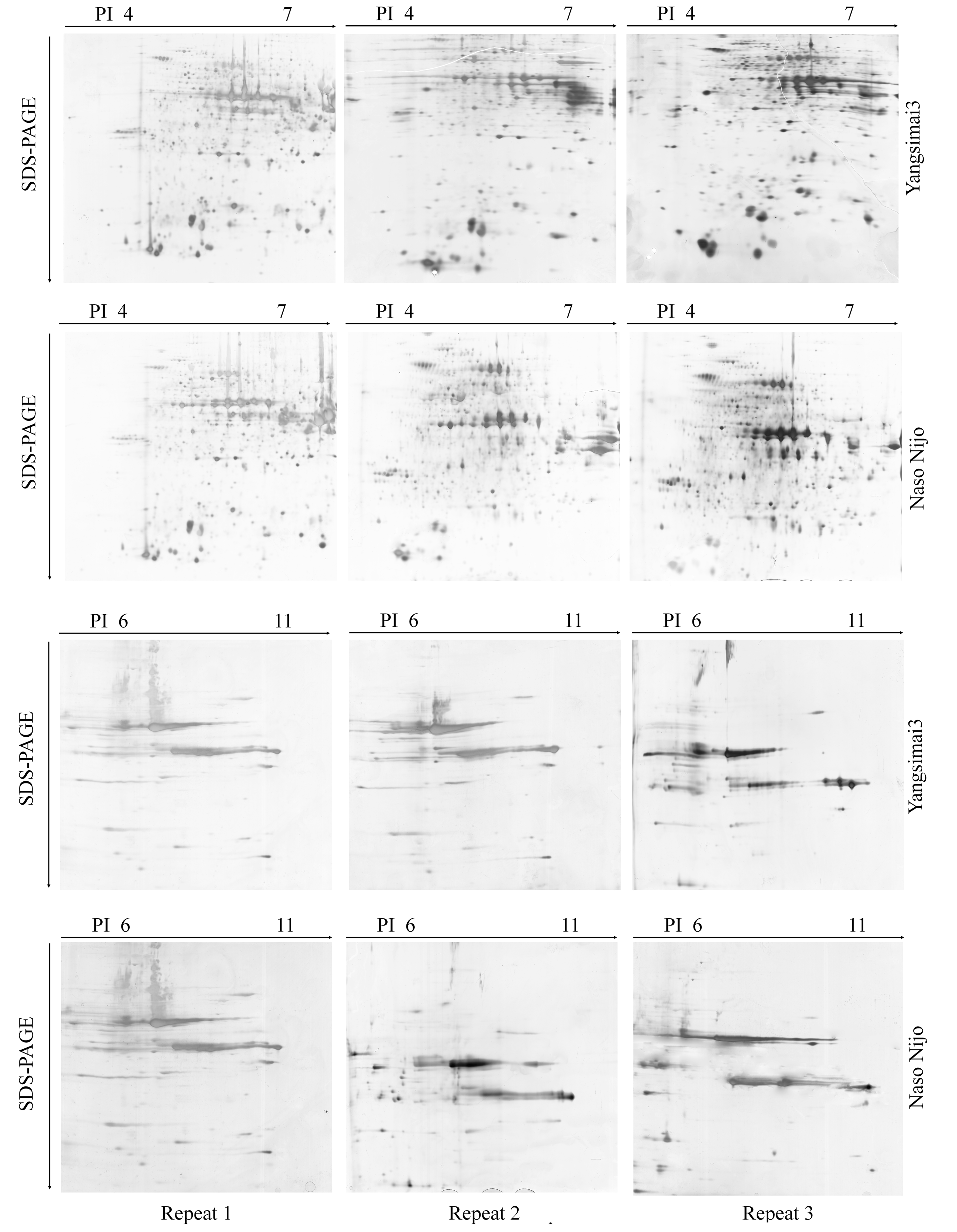

Supplement: FIGURE S2 — All the replicates 2-DE gels in the present study. [file Image_2.TIF]

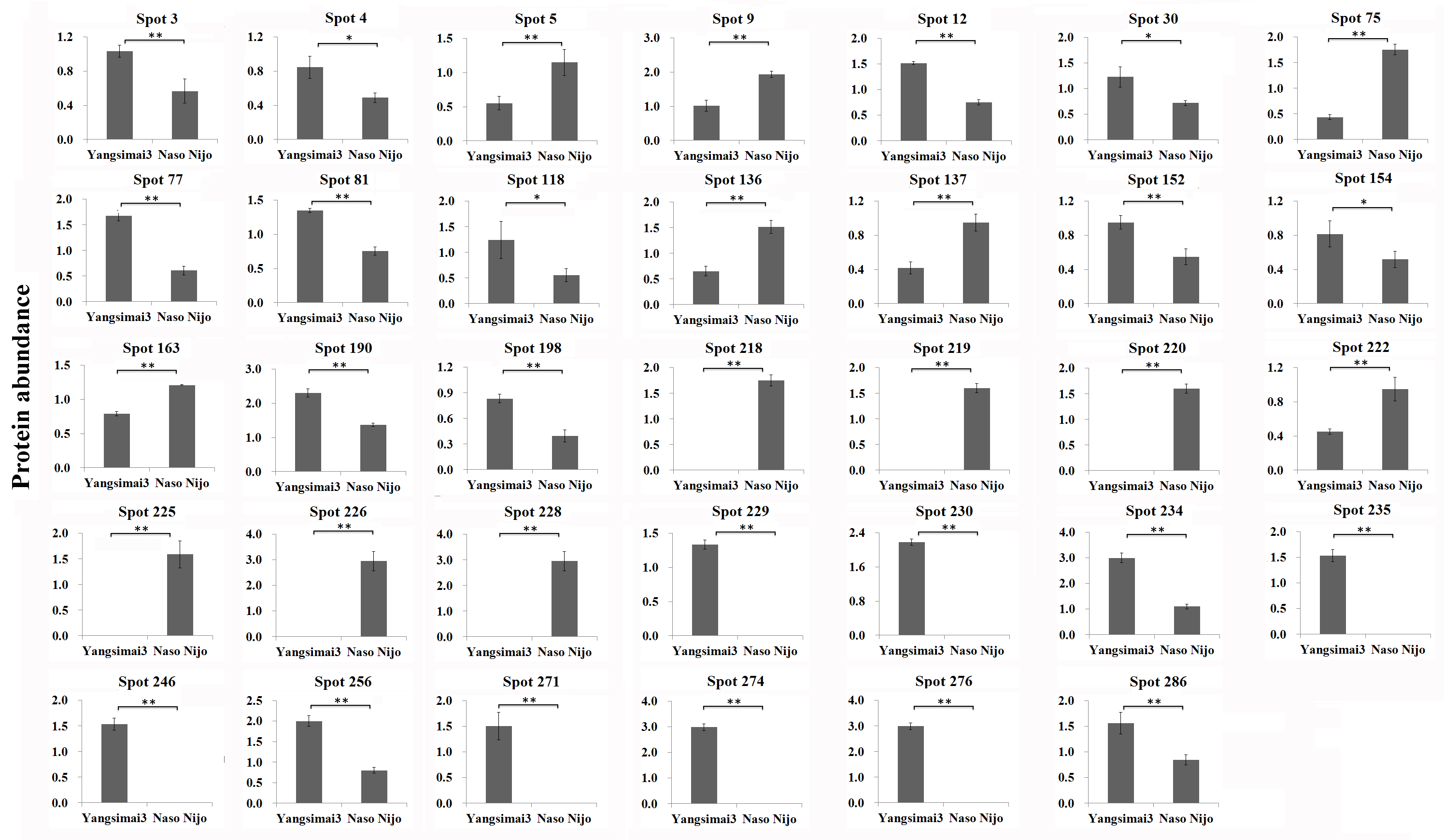

Supplement: FIGURE S3 — Expression patterns for differentially expressed protein spots. ∗p < 0.05, ∗∗p < 0.01. [file Image_3.TIF]
